# Humans and great apes share increased neocortical neuropeptide Y innervation compared to other haplorhine primates

**DOI:** 10.3389/fnhum.2014.00101

**Published:** 2014-02-28

**Authors:** Mary Ann Raghanti, Melissa K. Edler, Richard S. Meindl, Jessica Sudduth, Tatiana Bohush, Joseph M. Erwin, Cheryl D. Stimpson, Patrick R. Hof, Chet C. Sherwood

**Affiliations:** ^1^Department of Anthropology, School of Biomedical Sciences, Kent State UniversityKent, OH, USA; ^2^Department of Anthropology, The George Washington UniversityWashington, DC, USA; ^3^Fishberg Department of Neuroscience and Friedman Brain Institute, Icahn School of Medicine at Mount SinaiNew York, NY, USA; ^4^New York Consortium in Evolutionary PrimatologyNew York, NY, USA

**Keywords:** NPY, Broca's area, Wernicke's area, primate evolution

## Abstract

Neuropeptide Y (NPY) plays a role in a variety of basic physiological functions and has also been implicated in regulating cognition, including learning and memory. A decrease in neocortical NPY has been reported for Alzheimer's disease, schizophrenia, bipolar disorder, and depression, potentially contributing to associated cognitive deficits. The goal of the present analysis was to examine variation in neocortical NPY-immunoreactive axon and varicosity density among haplorhine primates (monkeys, apes, and humans). Stereologic methods were used to measure the ratios of NPY-expressing axon length density to total neuron density (ALv/Nv) and NPY-immunoreactive varicosity density to neuron density (Vv/Nv), as well as the mean varicosity spacing in neocortical areas 10, 24, 44, and 22 (Tpt) of humans, African great apes, New World monkeys, and Old World monkeys. Humans and great apes showed increased cortical NPY innervation relative to monkey species for ALv/Nv and Vv/Nv. Furthermore, humans and great apes displayed a conserved pattern of varicosity spacing across cortical areas and layers, with no differences between cortical layers or among cortical areas. These phylogenetic differences may be related to shared life history variables and may reflect specific cognitive abilities.

## Introduction

Neuropeptide Y (NPY), a 36-amino acid peptide, has an evolutionarily conserved sequence (Blomqvist et al., 1992), and is expressed at high levels throughout the central nervous system (Tatemoto et al., 1981; Adrian et al., 1983). The NPY that is expressed in the cerebral cortex derives from both intrinsic and extrinsic sources. NPY is often colocalized with GABA in cortical interneurons, and NPY-containing neurons of the olfactory bulb, nucleus of the tractus solitarius, and locus coeruleus send projections to innervate neurons of the cerebral cortex, spinal cord, and hypothalamus (Adrian et al., 1983; Allen et al., 1983; von Bohlen und Halbach and Dermietzel, 2006). The effects of NPY are mediated by at least four recognized receptor subtypes (Michel, 1991; Dumont et al., 1998). Its roles within the cerebral cortex include the regulation of blood flow and synaptic activity, and inhibition of neuronal excitability (Colmers and Bleakman, 1994; Cauli et al., 1997; Estrada and Defelipe, 1998; Bacci et al., 2002; Hamel et al., 2002). NPY is involved in regulating a variety of physiological functions, such as feeding behaviors, sleep regulation, and cardiovascular neuroendocrine functions, but has also been implicated in cognitive functions, including learning and memory (Rangani et al., 2012). In addition, decreased NPY concentrations are associated with conditions such as schizophrenia, depression, bipolar disorder, and Alzheimer's disease (Beal et al., 1986b; Kowall and Beal, 1988; Caberlotto and Hurd, 1999; Kuromitsu et al., 2001; Frederickson et al., 2007; Morales-Medina et al., 2010). Accordingly, evolutionary changes in the synthesis and innervation pattern of NPY may be present among primates related to cognitive and behavioral variability.

Neuroanatomical evidence indicates that the distribution and density of axons selectively expressing several neocortical neurotransmitters exhibit differences among primate species (Raghanti et al., 2008a,b,c). Humans and chimpanzees share increased neocortical innervation by dopaminergic and serotonergic axons and a different laminar pattern of cholinergic afferents selectively within prefrontal cortex (areas 9 and 32) relative to macaques, with no species differences evident in the primary motor cortex (area 4) (Raghanti et al., 2008a,b,c). In primates and other mammals, neocortical neurotransmitter innervation has been demonstrated to have species- and area-specific patterns (Morrison et al., 1982; Morrison and Foote, 1986; Lewis et al., 1987; Berger et al., 1991, 1992), raising the possibility that these systems may provide targets for the evolutionary modification of neuronal processing capacity. To understand the role of neurotransmitters in the evolution of the human brain, each system must be analyzed in a comparative context, so ultimately, broad quantitative comparative analyses of each cortical neurotransmitter system can be integrated and evaluated to reveal species-typical neurochemical phenotypes.

In the present study, we quantified the density of NPY-immunoreactive (ir) axons and varicosities in neocortical areas 10, 24, 44, and 22 among humans, African great apes, Old World monkeys, and New World monkeys. Cortical areas were chosen based on their roles in cognitive functions, including regions involved in language. The goal of the study was to determine if there was an evolutionary shift in cortical NPY innervation that might be associated with the emergence of cognitive or behavioral differences among haplorhine primates.

## Materials and methods

### Specimens

Brain samples from the left hemisphere of 46 individuals representing eight haplorhines species were used for this study and included New World monkeys (black-capped squirrel monkeys and tufted capuchins), Old World monkeys (moor macaques, pigtailed macaques, and olive baboons), African great apes (western lowland gorillas and common chimpanzees), and humans (see Table 1 for details). Brain weight was recorded for every specimen either at the time of necropsy or shortly afterwards. All individuals were adults and had no evidence of neuropathological alterations based on inspection of gross specimens. Sexes were balanced within species as much as possible given the opportunistic nature of brain collection. Human brain samples were provided by the Northwestern University Alzheimer's Disease Center Brain Bank. The human subjects exhibited no evidence of cognitive changes before death and all received a score of zero for the CERAD senile plaque grade (Mirra et al., 1991) and the Braak and Braak neurofibrillary tangle stage (Braak and Braak, 1991). Non-human primate brains were acquired from American Zoo and Aquarium-accredited zoos or research institutions and were maintained in accordance with each institution's animal care and use guidelines. All brains except those of the moor macaques were immersion-fixed in 10% buffered formalin for at least 7 days, transferred to a 0.1 M phosphate buffered saline solution containing 0.1% sodium azide, and stored at 4°C until processing. The moor macaques were perfused transcardially with 4% paraformaldehyde as part of unrelated experiments (Duan et al., 2003). Postmortem interval (PMI) did not exceed 17 h for any of the specimens. While all four cortical areas were available for the majority of specimens, some individuals were not represented for every area. All available materials were analyzed for neuron densities and axon length densities. A minimum of four individuals per species (except three for squirrel monkeys) was used to quantify varicosity densities.

**Table 1:** **Study sample**.

**Group**	**Species**	**Common name**	**Sex**	**Age**
Humans	Homo sapiens^*^	Human	M	54
	Homo sapiens^*^	Human	M	54
	Homo sapiens^*^	Human	M	56
	*Homo sapiens*	Human	F	40
	Homo sapiens^*^	Human	F	43
	*Homo sapiens*	Human	F	43
	*Homo sapiens*	Human	F	54
Great apes	Pan troglodytes^*^	Chimpanzee	F	44
	Pan troglodytes^*^	Chimpanzee	F	45
	Pan troglodytes^*^	Chimpanzee	M	25
	Pan troglodytes^*^	Chimpanzee	M	17
	Pan troglodytes^*^	Chimpanzee	M	19
	Gorilla gorilla^*^	Western lowland gorilla	M	13
	Gorilla gorilla^*^	Western lowland gorilla	M	21
	Gorilla gorilla^*^	Western lowland gorilla	M	42
	*Gorilla gorilla*	Western lowland gorilla	M	49
	Gorilla gorilla^*^	Western lowland gorilla	F	50
	*Gorilla gorilla*	Western lowland gorilla	F	55
Old World	Macaca nemestrina^*^	Pigtailed macaque	M	3
monkeys	Macaca nemestrina^*^	Pigtailed macaque	M	4
	Macaca nemestrina^*^	Pigtailed macaque	M	7
	Macaca nemestrina^*^	Pigtailed macaque	M	15
	Macaca nemestrina^*^	Pigtailed macaque	F	6
	Macaca nemestrina^*^	Pigtailed macaque	F	9
	Macaca nemestrina^*^	Pigtailed macaque	F	15
	Macaca maura^*^	Moor macaque	M	8
	Macaca maura^*^	Moor macaque	M	10
	Macaca maura^*^	Moor macaque	F	5
	*Macaca maura*	Moor macaque	F	7
	Macaca maura^*^	Moor macaque	F	7
	Macaca maura^*^	Moor macaque	F	8
	Papio anubis^*^	Baboon	M	5
	Papio anubis^*^	Baboon	M	7
	Papio anubis^*^	Baboon	M	9
	Papio anubis^*^	Baboon	M	10
	*Papio anubis*	Baboon	F	5
	Papio anubis^*^	Baboon	F	9
	Papio anubis^*^	Baboon	F	12
New World	Saimiri boliviensis^*^	Squirrel monkey	F	12
monkeys	Saimiri boliviensis^*^	Squirrel monkey	F	9
	Saimiri boliviensis^*^	Squirrel monkey	F	9
	Cebus apella^*^	Capuchin	M	15
	Cebus apella^*^	Capuchin	M	16
	Cebus apella^*^	Capuchin	F	12
	*Cebus apella*	Capuchin	F	17
	Cebus apella^*^	Capuchin	F	18

Age is in years, M, male; F, female.

* included in among-species analyses of ALv/Nv (i.e., all four cortical areas were represented).

### Sample processing

Prior to sectioning, specimens were cryoprotected in a series of sucrose solutions (10, 20, and 30%) until saturated. Brains were frozen in dry ice and cut in the coronal plane into 40 μm-thick sections using a Leica SM2000R freezing sliding microtome. Sections were placed into individual microcentrifuge tubes containing a freezer storage solution (30% of each distilled water, ethylene glycol, and glycerol and 10% 0.244 M phosphate-buffered saline) and numbered sequentially. Sections were stored at −20°C until further processing. Every tenth section was stained for Nissl substance with a solution of 0.5% cresyl violet. Nissl-stained sections were used to identify the cortical areas of interest based on cytoarchitecture.

### Identification of cortical areas

The identification of each cortical area was performed using descriptions from previous parcellations in a wide range of primate species, including galagos, New World monkeys, Old World monkeys, apes, and humans (Bailey et al., 1950; Rosabal, 1967; Preuss and Goldman-Rakic, 1991b; Watanabe-Sawaguchi et al., 1991; Vogt et al., 1995; Petrides and Pandya, 1999; Paxinos et al., 2000; Sherwood et al., 2003, 2004; Petrides, 2005; Schenker et al., 2008; Spocter et al., 2010). Because the cytoarchitecture of the areas of interest have not been described for all species analyzed in the present study, reports from a diverse array of primates were used to identify the cortical areas using Nissl-stained sections.

Broca's area (area 44) and its homolog have been described in humans (Amunts et al., 1999; Petrides, 2005), chimpanzees (Sherwood et al., 2003), and other great apes (Schenker, 2007). There has been considerable controversy over whether monkeys possess a homolog of human area 44. Petrides et al. (2005) addressed this question using both electrophysiological recordings and quantitative cytoarchitecture in macaque monkeys, demonstrating the presence of a cortical area that is topologically and cytoarchitecturally comparable to that of humans and which is involved in orofacial muscle movement. Coudé et al. (2011) also recently reported that voluntary vocalizations, but not emotional vocalizations, evoked the firing of neurons within the macaque homolog of Broca's area. Area 44 is characterized in each species as being dysgranular with a poorly developed layer IV and large pyramidal cells in the lower portion of layers III and V.

Wernicke's area and its homolog (area 22 or Tpt) is well described for humans (Fullerton and Pandya, 2007), chimpanzees (Spocter et al., 2010), macaques (Preuss and Goldman-Rakic, 1991a; Lewis and Van Essen, 2000; Fullerton and Pandya, 2007; Gannon et al., 2008), and galagos (Preuss and Goldman-Rakic, 1991a). Area Tpt is located in the caudal portion of the superior temporal gyrus and is readily distinguished by its well-developed layer II, a columnar appearance of pyramidal neurons in layers III and V and a thick layer IV (Galaburda and Pandya, 1982; Preuss and Goldman-Rakic, 1991a; Fullerton and Pandya, 2007; Gannon et al., 2008).

Both areas 10 and 24 are present in all haplorhine primate species (Brodmann, 1909; Allman et al., 2001; Semendeferi et al., 2001; Burman et al., 2006; Passingham and Wise, 2012). The anterior cingulate cortex (area 24) is involved in visceral, emotional, and cognitive processes including control of heart rate, blood pressure, vocalizations, and facial expressions (Smith, 1945; Jürgens and Ploog, 1970; Jürgens, 1998). This area also regulates emotional self-control (Damasio et al., 2000), and is activated during cognitively demanding tasks as well as intense drive states in humans (Paus et al., 1993, 1998; Bartels and Zeki, 2000; Bush et al., 2000). The cytoarchitecture of area 24 has been described in humans (Vogt et al., 1995; Öngür and Price, 2000; Öngür et al., 2003), macaques (Hof and Nimchinsky, 1992; Carmichael and Price, 1994; Dombrowski et al., 2001; Vogt et al., 2005), and in a study that compared the cytoarchitecture of humans to that of macaques (Petrides and Pandya, 1994). Area 24 is located in the anterior cingulate gyrus (Carmichael and Price, 1994; Vogt et al., 1995, 2005; Öngür et al., 2003) and the region sampled for this study was anterior to the genu of the corpus callosum, which is agranular.

Area 10 is involved in verbal and non-verbal episodic memory, reward-based decision making, and planning future actions (Buckner, 1996; Okuda et al., 1998; Daffner et al., 2000; Lepage et al., 2000; Burgess et al., 2007). Lesions restricted to area 10 in humans impair the ability to extract meaning from events, and limit creative thinking, artistic expression, and disrupt the ability to plan future actions. Semendeferi et al. (2001) assessed the homology of area 10 among humans, apes, and macaque monkeys. The authors found that area 10 of the frontal pole in human, bonobo, chimpanzee, and orangutan was homologous with the orbital sector of the frontal pole in gibbons. The orbitolateral component of the frontal pole in macaques most closely resembled area 10 of the other species (Semendeferi et al., 2001). Area 10 also has been described in the hamadryas baboon (Watanabe-Sawaguchi et al., 1991), squirrel monkey (Rosabal, 1967), and marmoset (Burman et al., 2006). Area 10 is a granular cortex with a thin layer II, wide layer III, and a sharp border between layer VI and white matter (Semendeferi et al., 2001).

### Immunohistochemistry

Every tenth section from each individual and cortical area was processed for NPY immunohistochemistry using the avidin-biotin-peroxidase method as described previously (Raghanti et al., 2013). Briefly, sections were pretreated for antigen retrieval by incubation in 0.05% citraconic acid (pH 7.4) at 85–90°C for 30 min. Endogenous peroxidase was quenched and sections were preblocked, pretreated, and incubated in a polyclonal primary antibody (Abcam, Cambridge, MA, catalog # ab30914) at a dilution of 1:5000 for 24 h at room temperature followed by 24 h at 4°C (Massoner et al., 2013; Raghanti et al., 2013). This antibody is derived from within residues 1 to the C-terminus of pig NPY. NPY has a high degree of sequence homology among vertebrates, with 22 of its 36 positions identical in all sequenced species (Larhammar et al., 1987; Larhammar, 1996), and 100% sequence homology in human, rabbit, rat, guinea pig, and alligator (O'Hare et al., 1988; Larhammar, 1996). After incubation in primary antibody, sections were incubated in a biotinylated secondary antibody (1:200) followed by the avidin-peroxidase complex (PK-6100, Vector Laboratories, Burlingame, CA). A 3,3′-diaminobenzidine-peroxidase (DAB) substrate with nickel enhancement was used as the chromogen (SK-4100, Vector Laboratories).

### Data acquisition

Quantitative analyses were performed using computer-assisted stereology with a system including an Olympus BX-51 photomicroscope equipped with a digital camera and StereoInvestigator software version 10 (MBF Biosciences, Williston, VT). Sampling parameters for each species and variable were determined using subsampling techniques to obtain a coefficient of error below 0.08 (Slomianka and West, 2005). Sampling areas (i.e., layers III and V-VI of each area) were outlined at low magnification (4×, N.A. 0.13). Total axon length was measured utilizing the SpaceBalls probe under Koehler illumination at 60x using the following equation: *L* = 2 * (v/a) * (Σ is) * 1/asf * 1/ssf * 1/tsf, where v/a is the ratio of sampling frame volume to probe surface area, Σis is the sum of the number of intersections between fibers and sampling hemispheres, asf (area sampling fraction; the fraction of the total area sampled) is the area of the counting frame divided by the total area of the reference space, section sampling fraction (ssf) is the number of sections analyzed divided by the total number of sections through the reference space, and tissue sampling fraction (tsf) is the sampling box height divided by mean mounted section thickness (Calhoun et al., 2004). Mean mounted section thickness was measured at every fifth sampling site. To measure axon length density (ALv), total axon length was divided by the planimetric measurement of the reference volume sampled.

Axonal varicosities, or synaptic boutons, are enlarged areas of an axon that contain release sites for neurotransmitters (Sutoo et al., 2001; Shepherd and Raastad, 2003), and were quantified as a putative measure of neuron-specific innervation to differentiate between axons that are directly innervating cells of a specific area vs. those that are *en passant*. Varicosities were defined as having a transverse diameter greater than 0.5 μm (Mechawar et al., 2000). Varicosity density (Vv) was quantified using the optical disector probe with a 100× (N.A. 1.4) objective lens. Vv was calculated as the sum of varicosities counted divided by the product of the disector and the volume of the disector (e.g., Sherwood et al., 2007). In addition to varicosity density, mean spacing of varicosities was calculated for each cortical layer and area for each species using the calculated estimates of NPY-ir Vv and ALv. Varicosity spacing has received attention as a putative measure of synaptic density (Shepherd et al., 2002; Shepherd and Raastad, 2003). Here, we aimed to evaluate the variability of varicosity spacing (i.e., mean spacing of varicosities in μm) to determine if this system is globally conserved across cortical regions within a species and among species. Nissl-stained sections were used to obtain neuron density (Nv) with a 60× (N.A. 1.35) objective lens. Nv was calculated in the same manner as Vv.

### Statistical analyses

The variables used to compare cortical NPY innervation among species were ALv/Nv, Vv/Nv, and mean varicosity spacing. The ratio of NPY-ir axon and varicosity density to neuron density allowed for comparison among species with divergent brain sizes. Factorial analyses of variance (ANOVA) with repeated-measures design were used to analyze differences among species for NPY-ir ALv/Nv, Vv/Nv, and mean varicosity spacing. Cortical area and layer were within-subjects factors and species was the between-subjects factor. Repeated-measures ANOVAs also were used to evaluate the uniformity of varicosity spacing within each species with both layer and area as within-subjects factors. Fisher's LSD *post hoc* analyses were employed to evaluate significant findings. Nonparametric Spearman's correlation coefficient was used to assess whether PMI affected the intensity of immunohistochemical staining in humans. Exact PMIs were not known for other species. Additionally, Spearman's correlation was used to test the effect of age on cortical NPY innervation within each species and the relationship between ALv/Nv and Vv/Nv. Independent *t*-tests were used to examine differences between sexes. Standard linear regression was used to determine if differences in cortical ALv or Vv were associated with variation in brain mass or encephalization quotient. Brain masses were obtained for each specimen before processing. Because body mass was not available for all individuals, encephalization quotients were taken from Jerison (1973). Neither body mass nor encephalization quotients were available for moor macaques and were omitted from these analyses. Finally, an intraclass correlation coefficient was used to assess inter-observer reliability for quantitative stereological data. Periodically, inter-observer reliability was evaluated utilizing an audit feature within the StereoInvestigator software. The level of significance (α) was set at 0.05 for all statistical tests.

## Results

**Figure 1:**
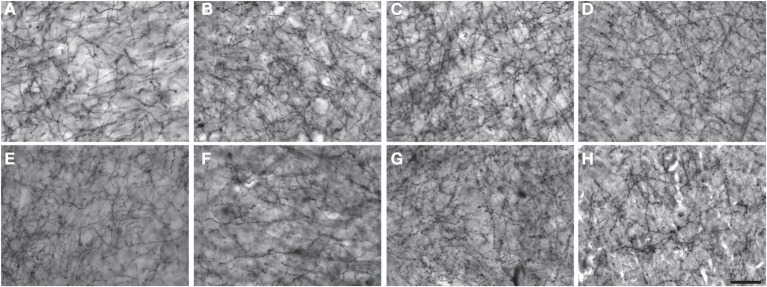
**Photomicrographs of NPY immunostaining in layer III of area 44 in (A) squirrel monkey, (B) capuchin, (C) moor macaque, (D) pigtailed macaque, (E) baboon, (F) gorilla, (G) chimpanzee, and (H) human.** Scale bar = 50 μm.

**Figure 2:**
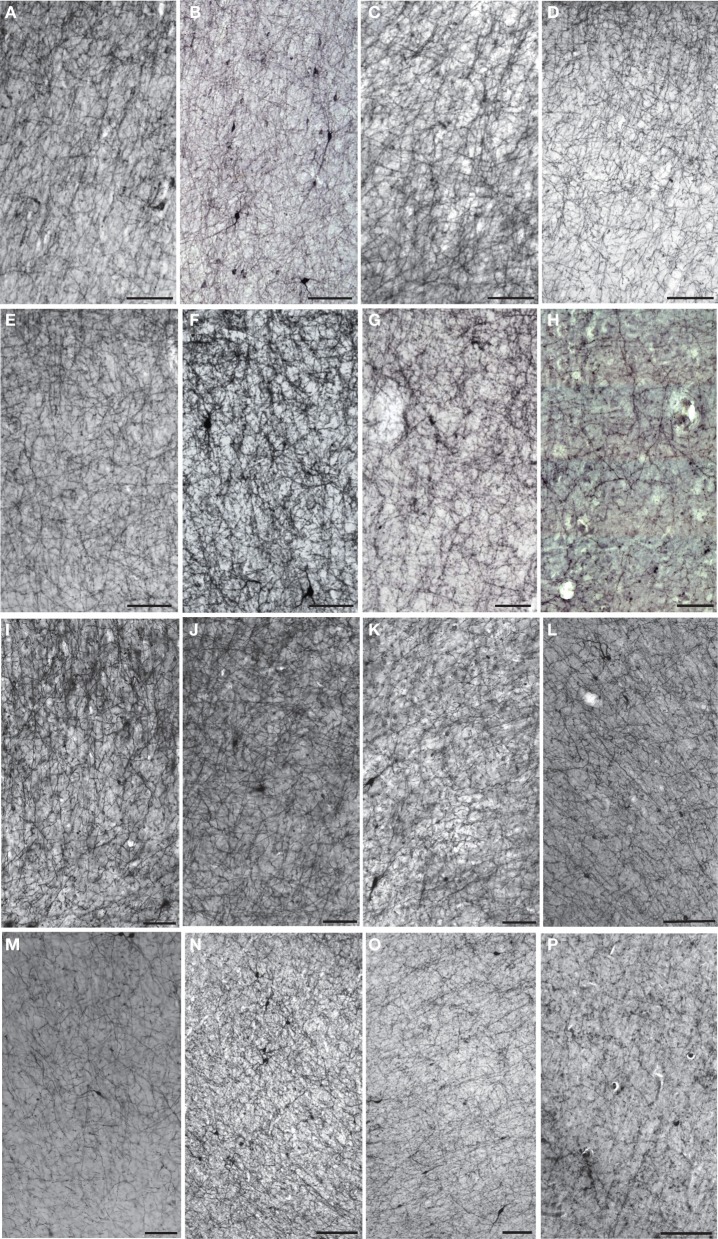
**Low-powered photomicrographs of NPY immunostaining in layer III (A–H) and layers V–VI (I–P) of area 10. (A,I)** squirrel monkey, **(B,J)** capuchin, **(C,K)** moor macaque, **(D,L)** pigtailed macaque, **(E,M)** baboon, **(F,N)** gorilla, **(G,O)** chimpanzee, **(H,P)** human. Scale bars = 100 μm.

**Figure 3:**
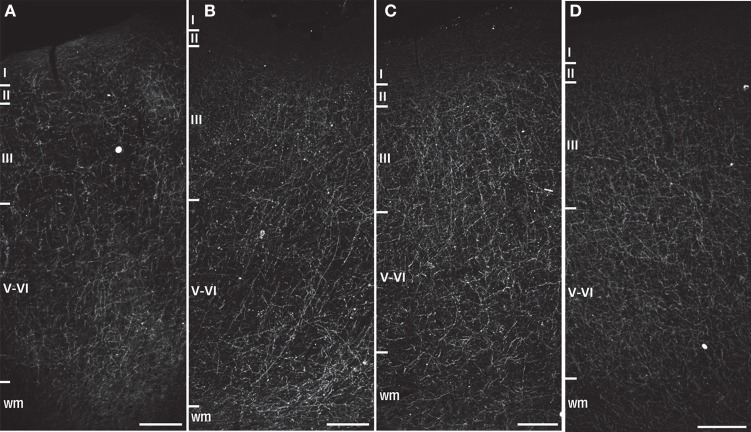
**Darkfield photomicrographs of NPY immunostaining in area 24 of (A) capuchin, (B) baboon, (C) gorilla, and (D) chimpanzee.** Roman numerals indicate cortical layers, wm, white matter. Scale bars = 250 μm.

Figures 1–3 provide examples of cortical NPY immunostaining for each species. The mean number of sampling sites for each layer/area/individual was 109.8 ± 25.4 for Nv (total sampling sites = 39,730; total neurons counted = 238,487), 86.4 ± 11.7 for ALv (total sampling sites = 33,869; total intersections counted = 234,583), and 68.5 ± 6.2 for Vv (total sampling sites = 19,433; total varicosities counted = 148,548). The mean coefficient of error was 0.04 ± 0.01 for Nv and 0.05 ± 0.01 for Vv (Schmitz and Hof, 2000). The intraclass correlation coefficient for inter-observer reliability was 0.86 (*n* = 24, *p* < 0.01).

### Postmortem interval

We examined the effect of PMI on our measures of cortical NPY in humans, where the most precise data on PMI were available. In humans, PMI was not correlated with NPY-ir ALv/Nv in layers III or V-VI in area 10 (Spearman's rho = −0.23, *p* = 0.66; −0.12, *p* = 0.83), area 24 (Spearman's rho = −0.21, *p* = 0.74; 0.15, *p* = 0.81), or area 22 (Spearman's rho = 0.56, *p* = 0.61; 0.31, *p* = 0.61). PMI was not correlated with NPY ALv/Nv in layer III of area 44 (Spearman's rho = 0.56, *p* = 0.32), but a significant correlation existed within layers V-VI (Spearman's rho = −0.81, *p* = 0.03). Given that 87.5% of possible correlations were not significant and PMI did not exceed 17 h for any individual, PMI is not considered a confounding variable for this study.

### Sex

Independent samples *t*-tests were used to determine possible differences in NPY-ir ALv/Nv or Vv/Nv between males and females in each species except squirrel monkeys, as only females were available from this species. No sex differences were detected in capuchins, pigtailed macaques, baboons, chimpanzees, or gorillas. In humans, females exhibited a denser ALv/Nv relative to males only for layers V-VI of area 22 [*t*_(3)_ = −3.51, *p* = 0.04]. There was also a sex difference for layer III of area 44 in moor macaques [*t*_(4)_ = −3.32, *p* = 0.03], with females having a denser ALv/Nv. Given these results, the overall effect of sex on cortical NPY innervation was not substantial.

### Age

To determine if there was a significant effect of age on NPY-ir ALv/Nv and Vv/Nv, each species was analyzed separately, for a total of 64 correlation analyses per measure. Age was not correlated with NPY ALv/Nv in any layer or area for non-human species. In humans, only layer III of area 22 showed an effect of age (Spearman's rho = −0.95, *p* = 0.01). For Vv/Nv, two significant correlations were found for pigtailed macaques (layer III of area 44, Spearman's rho = −0.90, *p* < 0.01; layers V-VI of area 22, *p* = −1.00, *p* < 0.01) and one for capuchins (layer III of area 10, Spearman's rho = 1.00, *p* < 0.01). Age within the present was not a significant variable as only 0.02% (ALv/Nv) and 0.05% (Vv/Nv) of the possible correlations were significant.

### Brain mass and encephalization quotient

NPY-ir ALv and Vv were each regressed on brain mass and encephalization quotient. There was a significant relationship between ALv and brain mass [*F*_(1, 343)_ = 28.48, *p* < 0.01, *R*^2^ = 0.08], but not Vv and brain mass [*F*_(1, 289)_ = 1.66, *p* = 0.20, *R*^2^ = 0.006). Neither measure was significant when regressed on encephalization quotient [ALv: *F*_(1, 296)_ = 2.70, *p* = 0.10, *R*^2^ = 0.009; Vv: *F*_(1, 250)_ = 0.05, *p* = 0.83, *R*^2^ = 0.00].

### Among-species analyses

**Figure 4:**
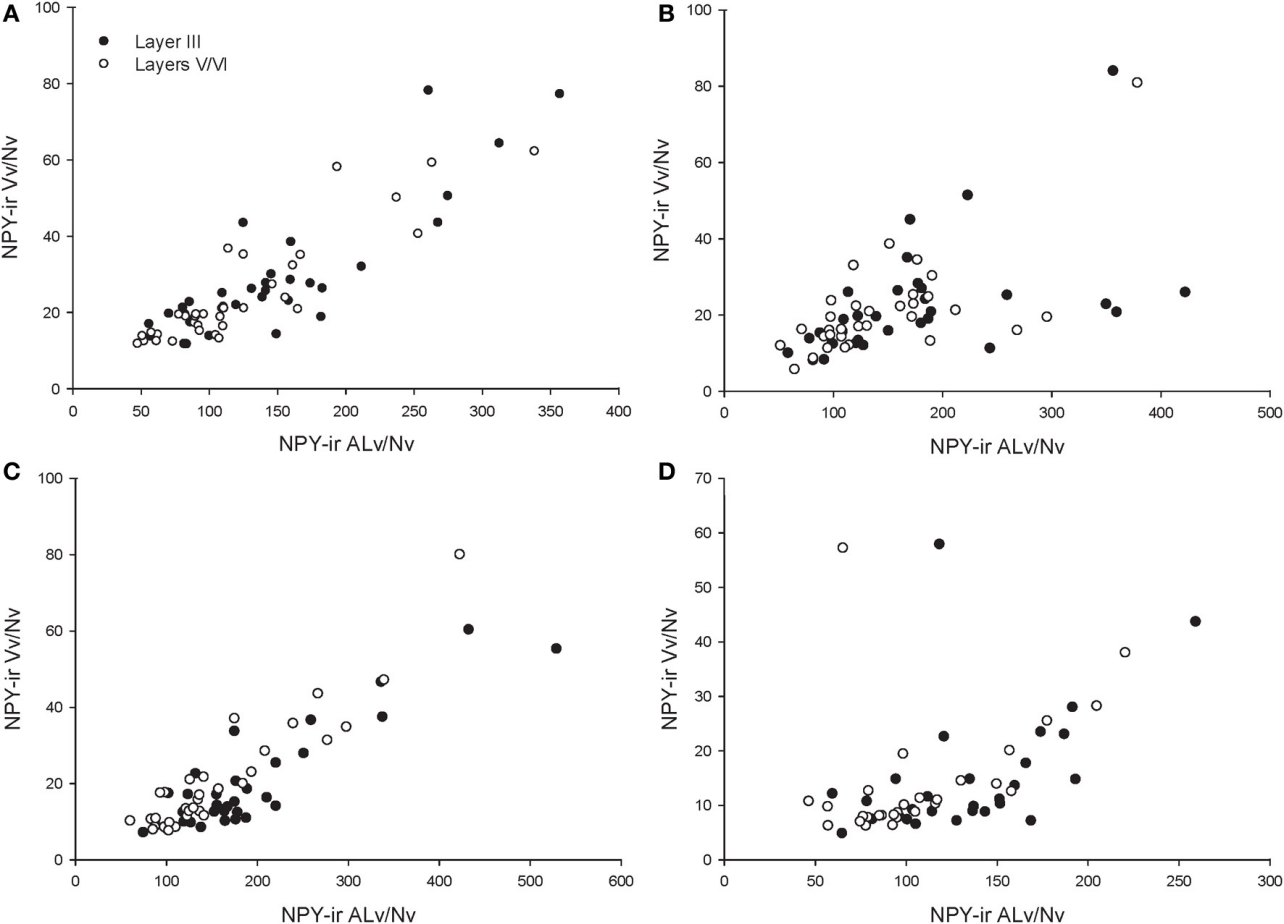
**Correlations between NPY-ir ALv/Nv and Vv/Nv for (A) area 10, (B) area 24, (C) area 44, and (D) area 22.** The outlier in **(B)** and **(D)** is one chimpanzee male.

**Figure 5:**
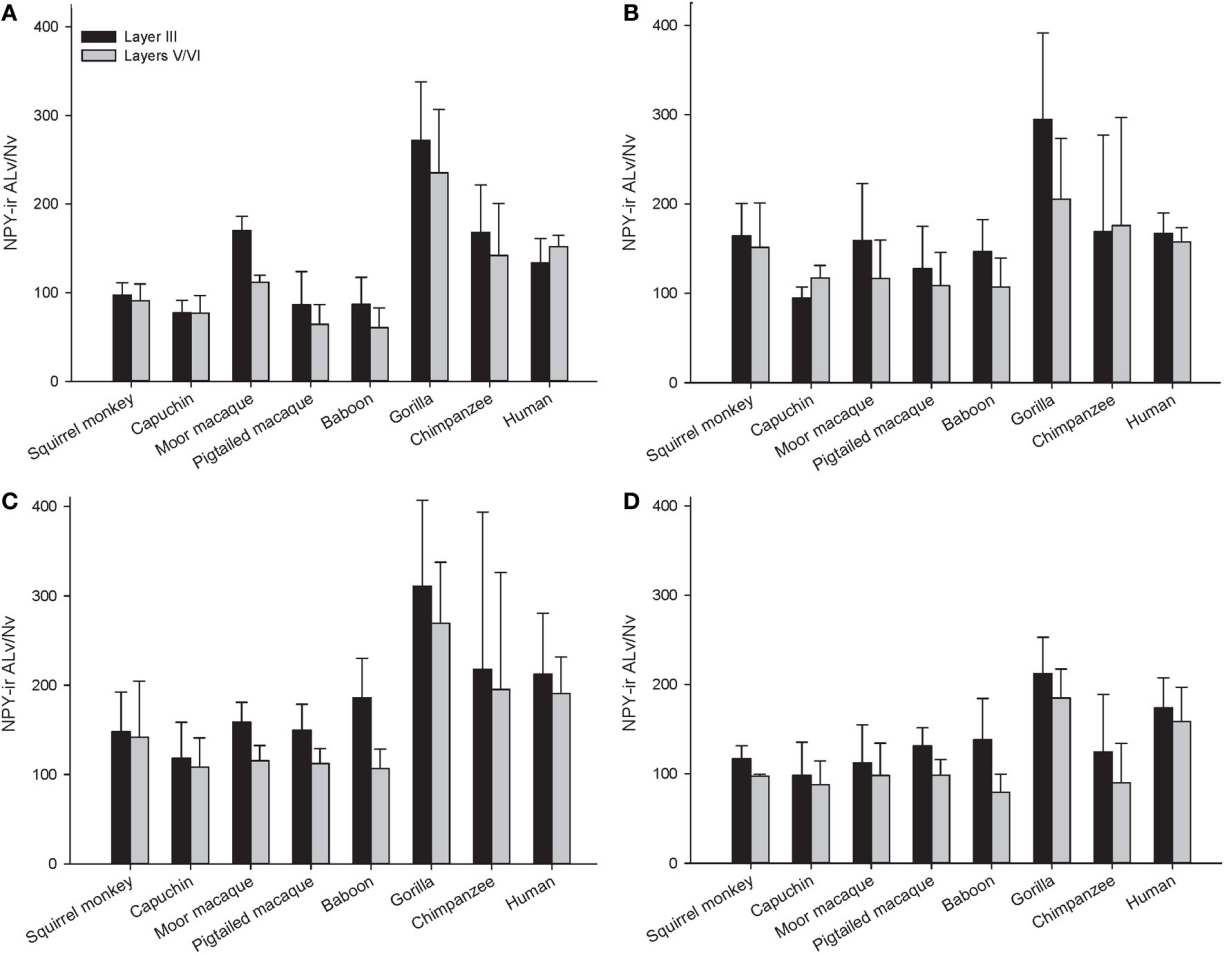
**NPY-ir ALv/Nv for each species for (A) area 10, (B) area 24, (C) area 44, and (D) area 22.** Means are shown by bars. Error bars indicate standard deviation.

**Figure 6:**
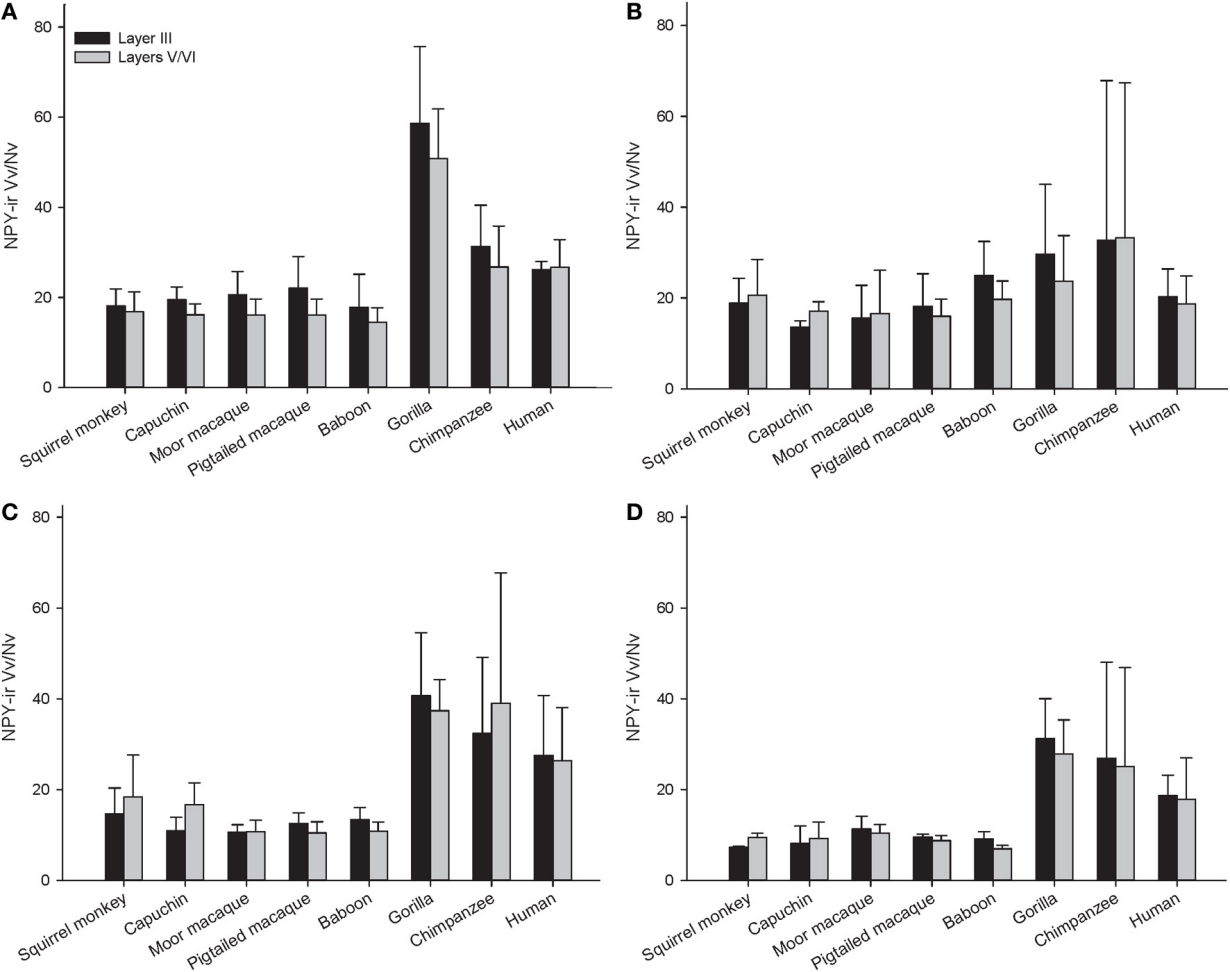
**NPY-ir Vv/Nv for each species in (A) area 10, (B) area 24, (C) area 44, and (D) area 22.** Means are shown by bars. Error bars indicate standard deviation.

**Figure 7:**
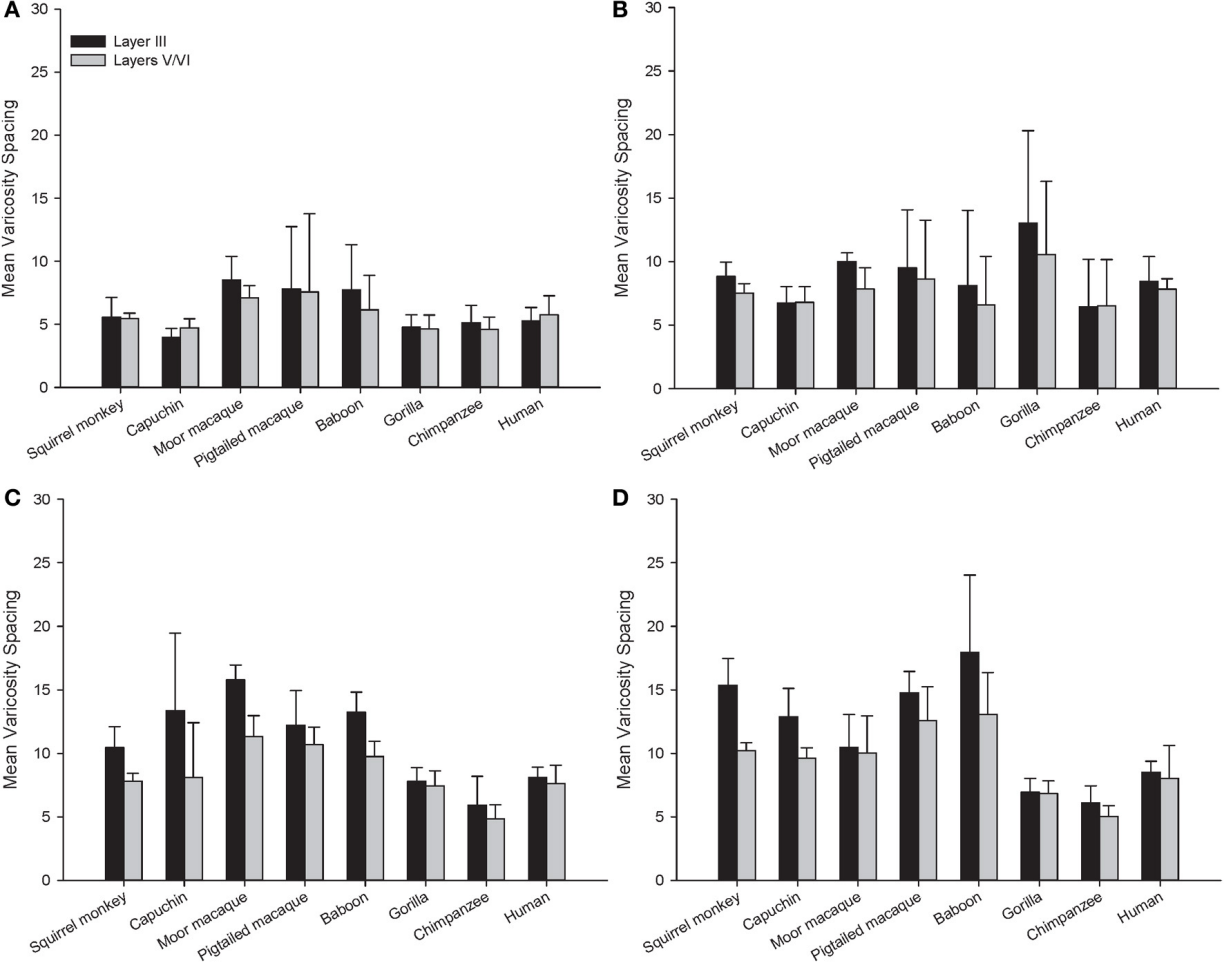
**Varicosity spacing in μm for each species in (A) area 10, (B) area 24, (C) area 44, and (D) area 22.** Means are shown by bars. Error bars indicate standard deviation.

Across the entire primate sample, NPY-ir ALv/Nv was significantly correlated with Vv/Nv in layers III and V-VI in all cortical areas (Spearman's rho, all *p*'s < 0.05, Figure 4). In some individuals, not every cortical area was represented due to tissue damage during brain extraction or unavailability of tissue. Therefore, analyses were restricted to individuals in which all four cortical areas were represented (see Table 1). Initial repeated-measures ANOVAs revealed no differences among species within the phylogenetic groups of New World monkeys, Old World monkeys, and hominids (great apes and humans) for ALv/Nv (Figure 5), Vv/Nv (Figure 6), and varicosity spacing (Figure 7). Thus, species were collapsed into these categories for further analysis.

**Figure 8:**
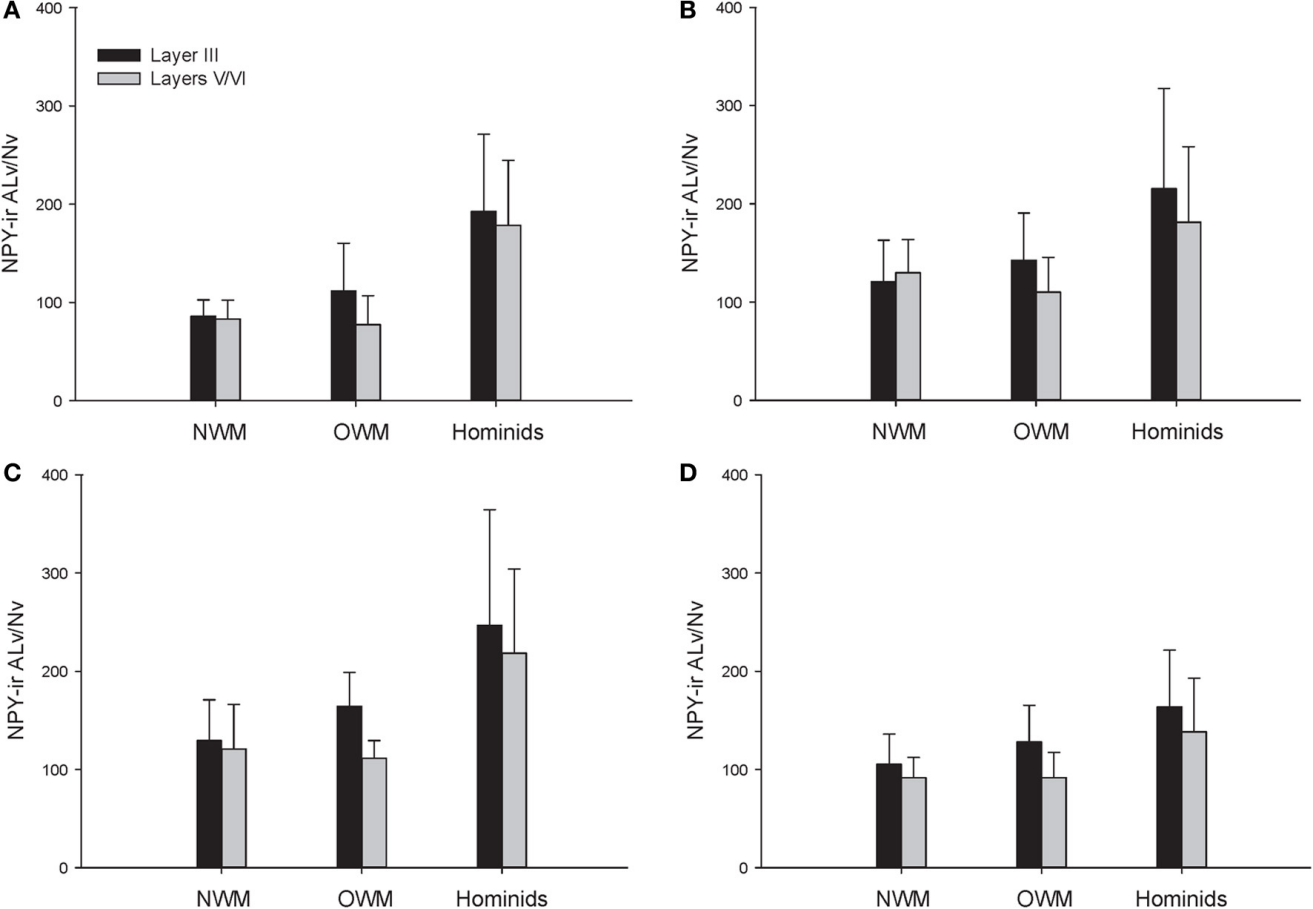
**NPY-ir ALv/Nv collapsed into phylogenetic groups for (A) area 10, (B) area 24, (C) area 44, and (D) area 22.** OWM, Old World monkeys; NWM, New World monkeys. Means are shown by bars. Error bars indicate standard deviation.

A repeated-measures ANOVA was used to analyze ALv/Nv among New World monkeys, Old World monkeys, and hominids (Figure 8). There were significant main effects of phylogenetic group [*F*_(2, 34)_ = 10.34, *p* < 0.01], cortical area [*F*_(3, 102)_ = 8.24, *p* < 0.01], and layer[*F*_(1, 34)_ = 14.75, *p* < 0.01]. Additionally, the interaction between layer and phylogenetic group was significant [*F*_(2, 34)_ = 6.32, *p* < 0.01]. The interaction terms of area by phylogenetic group, area by layer, and the three-way interaction of area by layer by phylogenetic group were not significant (all *p*'s > 0.05). Fisher's LSD *post-hoc* analyses revealed that hominids had significantly higher ALv/Nv relative to both New World monkeys and Old World monkeys (all *p*'s < 0.01). No differences were detected between the monkey groups (*p* = 0.50).

**Figure 9:**
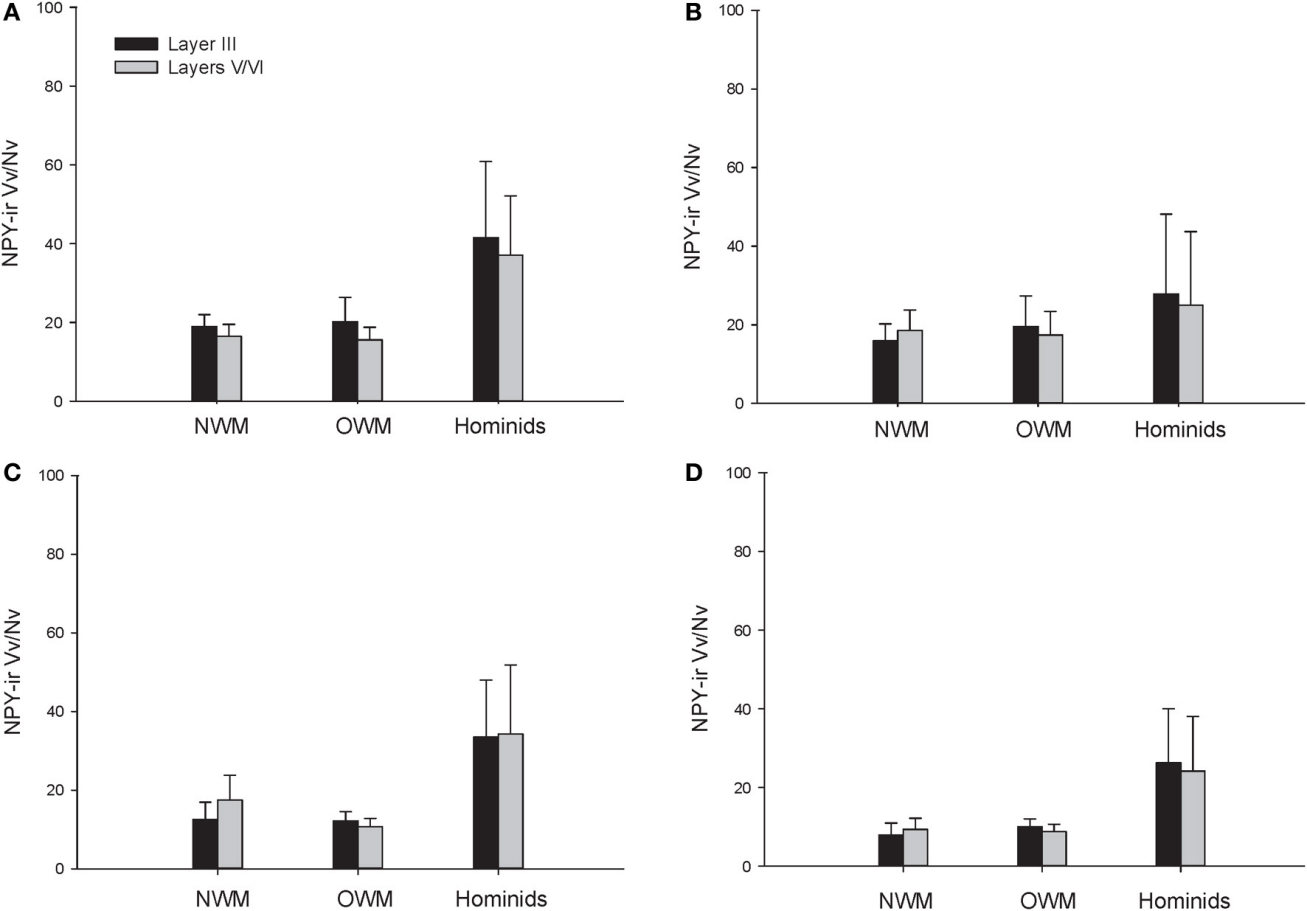
**NPY-ir Vv/Nv collapsed into phylogenetic groups for (A) area 10, (B) area 24, (C) area 44, and (D) area 22.** OWM, Old World monkeys; NWM, New World monkeys. Means are shown by bars. Error bars indicate standard deviation.

The repeated measures ANOVA for Vv/Nv (Figure 9) revealed significant main effects of phylogenetic group [*F*_(2, 28)_ = 24.73, *p* < 0.01] and area [*F*_(3, 84)_ = 4.94, *p* < 0.01]. The main effect of layer [*F*_(1, 28)_ = 0.87, *p* = 0.36] and interaction terms were not significant (all *p*'s > 0.05). Fisher's LSD *post-hoc* analyses demonstrated that hominids had significantly higher Vv/Nv relative to both New World monkeys and Old World monkeys (all *p*'s < 0.05). No differences were detected between the monkey groups (*p* = 0.56).

**Figure 10:**
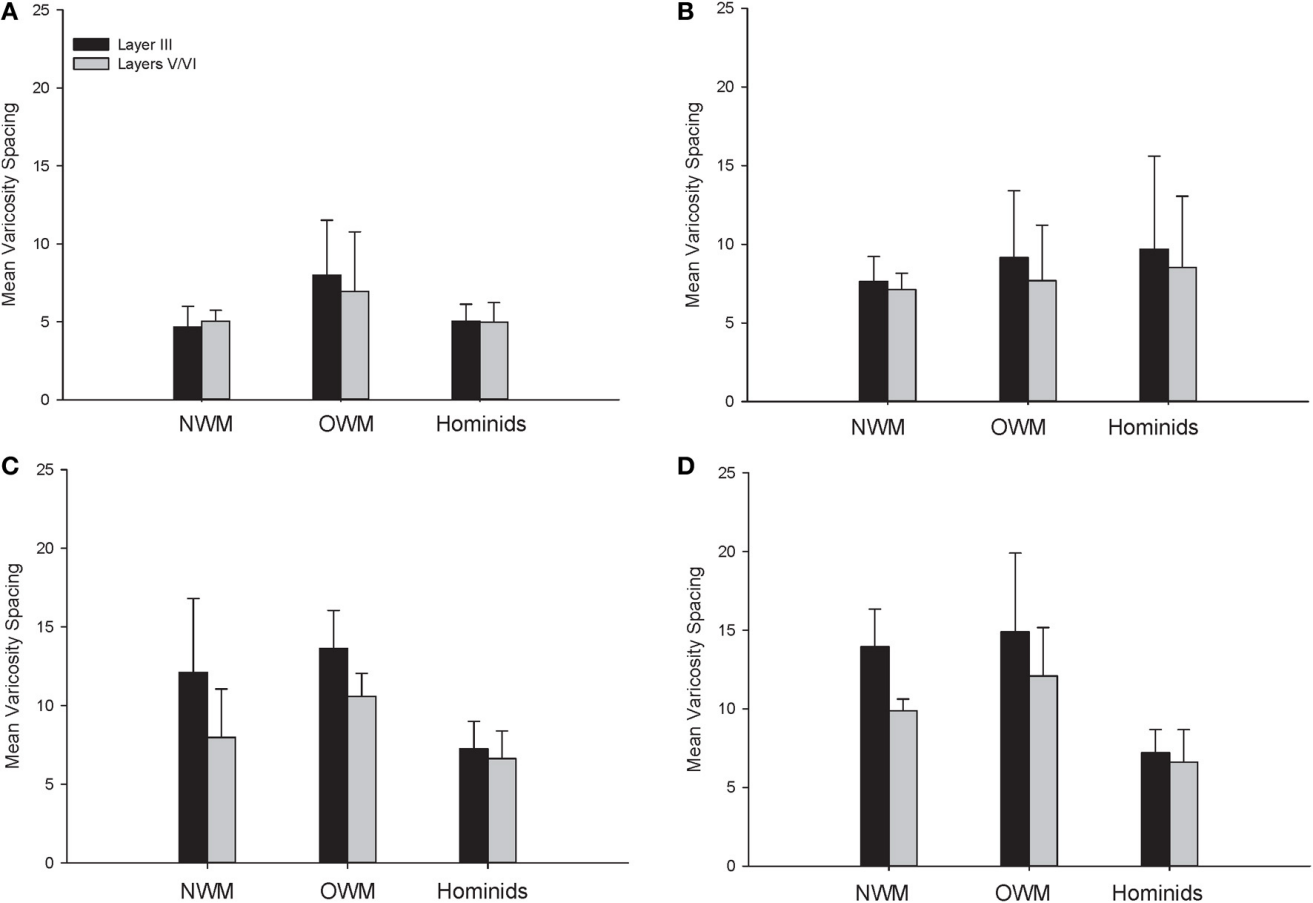
**NPY-ir varicosity spacing collapsed into phylogenetic groups for (A) area 10, (B) area 24, (C) area 44, and (D) area 22.** OWM, Old World monkeys; NWM, New World monkeys. Means are shown by bars. Error bars indicate standard deviation.

For varicosity spacing (Figure 10), there were significant main effects of phylogenetic group [*F*_(2, 28)_ = 17.35, *p* < 0.01], area [*F*_(3, 84)_ = 18.19, *p* < 0.01] and layer [*F*_(1, 28)_ = 76.19, *p* < 0.01]. The interaction of area by phylogenetic group [*F*_(6, 27)_ = 3.07, *p* < 0.05], layer by phylogenetic group [*F*_(2, 27)_ = 10.53, *p* < 0.01], and layer by area [*F*_(3, 27)_ = 10.47, *p* < 0.01] were significant. The three-way interaction of phylogenetic group by area by layer was not statistically significant [*F*_(6, 84)_ = 1.97, *p* = 0.08]. *Post-hoc* analyses showed that hominids had smaller inter-varicosity spacing relative to both New and Old World monkeys in area 22 (*p* = 0.03 and 0.001, respectively). Hominids also had a lower varicosity spacing relative to Old World monkeys in area 44 (*p* = 0.01). Furthermore, hominids displayed a lower mean varicosity spacing in layer III relative to the Old World monkeys (*p* = 0.02). No other significant differences were detected.

### Within-species analysis of varicosity spacing

Varicosity spacing was analyzed within each species to determine variability among cortical areas and layers using repeated-measures ANOVAs. A summary of main effects and interaction are listed in Table 2.

**Table 2:** **Summary of main effects (area and layer) and interaction term for the within-species repeated-measures ANOVA for varicosity spacing**.

	**Area**	**Layer**	Area * Layer
Squirrel monkey	*F*_(3, 6)_ = 34.15, *p* < 0.01^*^	*F*_(1, 6)_ = 21.43, *p* = 0.04^*^	*F*_(3, 6)_ = 3.59, *p* = 0.09
Capuchin	*F*_(3, 9)_ = 5.58, *p* = 0.02^*^	*F*_(1, 9)_ = 20.01, *p* = 0.02^*^	*F*_(3, 9)_ = 14.17, *p* < 0.01^*^
Moor macaque	*F*_(3, 9)_ = 6.29, *p* = 0.01^*^	*F*_(1, 9)_ = 79.83, *p* < 0.01^*^	*F*_(3, 9)_ = 6.40, *p* = 0.01^*^
Pigtailed macaque	*F*_(3, 12)_ = 4.01, *p* = 0.03^*^	*F*_(1, 12)_ = 66.76, *p* < 0.01^*^	*F*_(3, 12)_ = 0.54, *p* = 0.66
Baboon	*F*_(3, 9)_ = 7.43, *p* = 0.01^*^	*F*_(1, 9)_ = 23.98, *p* = 0.02^*^	*F*_(3, 9)_ = 2.26, *p* = 0.15
Gorilla	*F*_(3, 6)_ = 0.34, *p* = 0.80	*F*_(1, 6)_ = 23.38, *p* = 0.04^*^	*F*_(3, 6)_ = 0.95, *p* = 0.47
Chimpanzee	*F*_(3, 9)_ = 0.10, *p* = 0.96	*F*_(1, 9)_ = 1.52, *p* = 0.31	*F*_(3, 9)_ = 1.58, *p* = 0.26
Human	*F*_(3, 9)_ = 3.98, *p* = 0.07	*F*_(1, 9)_ = 0.71, *p* = 0.49	*F*_(3, 9)_ = 0.68, *p* = 0.60

Significant results (p < 0.05) are indicated by asterisks.

For squirrel monkey, *post-hoc* tests showed that area 22 had higher varicosity spacing relative to the other cortical areas, with area 10 having the lowest varicosity spacing (all *p*'s < 0.05). No significant differences between layers were detected (all *p*'s > 0.05). Capuchin varicosity spacing was higher in area 22 relative to the other areas and was lower in layers V-VI relative to layer III within areas 44 and 22 (all *p*'s > 0.05).

*Post-hoc* analyses for moor macaques showed that areas 44 and 22 had higher varicosity spacing relative to areas 10 and 24, and layers V/VI displayed lower varicosity spacing than layer III in areas 24 and 44 (all *p*'s < 0.05). In pigtailed macaques, there was increased spacing of varicosities in area 22 relative to area 10 (*p* = 0.04). Distance between varicosities was lower for layer V-VI relative to layer III (*p* = 0.02). For baboon, area 22 had higher varicosity spacing relative to areas 10 and 24 (*p* = 0.02 for each). *Post-hoc* analyses did not detect a significant difference between layers III and V/VI.

*Post-hoc* analyses for gorillas did not detect a significant difference between layers (*p* = 0.13). No significant differences were detected in either the chimpanzee or human.

## Discussion

The present report provides a broad quantitative comparative analysis of cortical NPY-ir axon and varicosity densities among haplorhine primate species. NPY is involved in a wide variety of physiological functions as well as cognitive processes, such as learning and memory, and deficits in NPY are associated with neuropathological conditions that exhibit decreases in intellectual abilities (Beal et al., 1986a; Kowall and Beal, 1988; Caberlotto and Hurd, 1999; Kuromitsu et al., 2001; Frederickson et al., 2007; Rangani et al., 2012). Our results demonstrated that humans and African great apes (i.e., hominids) shared a significant increase in cortical NPY-ir axons and varicosities relative to Old World monkeys and New World monkeys, as measured by NPY-ir ALv/Nv and Vv/Nv. Vv/Nv putatively represents a fine-grained measure of innervation as neurotransmitters are released from varicosities. Interestingly, the differences between hominids and monkeys are more apparent in this measure relative to ALv/Nv (see Figures 5, 6, 8, and 9). Although the overall effect of phylogenetic group (i.e., hominid vs. Old World and New World monkeys) was significant, area 24 did not appear dramatically different among the species analyzed here for either ALv/Nv or Vv/Nv. It is notable, however, that we did not find an increase in cortical NPY input for humans relative to the African great apes, chimpanzees and gorillas.

Increased NPY afferents to cortical regions, such as areas 10, 22, and 44, in humans and great apes could contribute to cortical processing by mediating synaptic inhibition, glutamatergic output, and an increase in cerebral blood flow. Interestingly, glutamatergic gene and protein expression are altered in the frontal pole of hominids relative to other primate species (Muntané et al., 2014), and these modifications may have co-evolved with increased cortical NPY. The most dramatic and consistent phylogenetic differences observed within the current data set were within the language areas (44 and 22), where humans and great apes exhibited higher NPY-ir Vv/Nv (see Figure 6); however, humans clearly deviate from apes in language proficiency. Ape language training experiments have required extensive effort to teach subjects to acquire a limited vocabulary and rudimentary syntax as compared to human children (Nowak and Komarova, 2001; Hauser et al., 2002). In addition to a shared capacity to learn certain aspects of symbolic language, humans and great apes also exhibit increased capacity for behavioral inhibition (Beran and Evans, 2006; Evans and Beran, 2007a,b), cultural transmission of knowledge (Boesch, 1993), episodic memory (Martin-Ordas et al., 2013), and an understanding of the attentional states of others (Tempelmann et al., 2011). Likewise, great apes consistently outperform other non-human primates in several cognitive tests, including mirror self-recognition, tool use, deception, and complex manipulation (Gallup, 1970; Tomasello et al., 2003; Deaner et al., 2006).

NPY may protect cortical neurons from glutamate excitotoxicity by inhibiting glutamate release (Decressac and Barker, 2012). NPY also protects cortical neurons against toxicity mediated by the amyloid β peptide, potentially by increasing the synthesis and release of nerve growth factor (Croce et al., 2012). In addition, NPY plays a significant role in neurogenesis (Decressac and Barker, 2012). It could be argued that these functions of NPY are critical to meet the demands of enlarged brains. However, neocortical NPY innervation density in our samples was not associated with encephalization quotient, and only 0.06–7.7% of the variance in measures of NPY innervation could be explained by brain size. Whereas brain mass varied by six-fold among the monkeys (squirrel monkey to baboon), there were no significant differences among these species in NPY innervation. Similarly, human brains are approximately three times larger than chimpanzees and gorillas, yet NPY density did not vary significantly among hominid species. This suggests that that the increase observed in hominids is not simply associated with encephalization, but rather represents a significant change that occurred in the development of NPY cortical innervation in the stem ancestral ape lineage.

The present analysis of NPY-ir varicosity spacing revealed interesting differences within each species. Notably, there was a conservation of varicosity spacing for humans, chimpanzees, and gorillas across cortical areas and between layers III and V/VI. For both New and Old World monkeys, there was higher varicosity spacing in area 22. There was also decreased varicosity spacing in layers V/VI relative to layer III for both macaque species and the capuchin monkey.

In an earlier study, we reported significant differences among haplorhine primate species in the density of cortical NPY-ir neurons in area 22 that did not clearly conform to a phylogenetic branching pattern (Raghanti et al., 2013). Interestingly, NPY-ir cortical neurons are one of the first affected neuron populations in Alzheimer's disease, suggesting a unique vulnerability among interneurons that are known to be rather resistant to the disease (Beal et al., 1986b; Kowall and Beal, 1988; Hof and Morrison, 1991; Hof et al., 1991, 1993). In our prior report, we found squirrel monkeys possessed the highest percentage of NPY-ir cortical neurons; however, that increase in NPY-ir cortical neurons was not associated with an increased NPY-ir ALv/Nv or Vv/Nv, as illustrated by the present data set. Overall, comparison of the density of NPY-ir cortical neurons with the results presented here indicates that the density of NPY-ir axons or varicosities is not correlated with the density of NPY-ir neurons within the cortex.

Our results indicate that humans and great apes share a significant increase in cortical NPY ALv/Nv and Vv/Nv relative to monkey species with a conserved pattern of NPY-ir varicosity spacing. The increase in cortical NPY innervation may contribute to the differences in behavioral and cognitive flexibility observed in hominids relative to other primates. Alternatively, this phylogenetic difference may be due to the increased lifespans observed within these lineages, since NPY peptide mediates neurodegenerative damage caused by glutamate turnover and amyloid β toxicity. Further research is necessary to determine the role of NPY within cortical language circuits and other executive functions and whether this difference in NPY innervation between hominids and monkeys extends to cortical regions that are not directly associated with cognition.

### Conflict of interest statement

The authors declare that the research was conducted in the absence of any commercial or financial relationships that could be construed as a potential conflict of interest.
